# High-Density Lipoprotein – A Hero, a Mirage, or a Witness?

**DOI:** 10.3389/fcvm.2014.00009

**Published:** 2014-11-14

**Authors:** Dmitri Sviridov

**Affiliations:** ^1^Baker IDI Heart and Diabetes Institute, Melbourne, VIC, Australia

**Keywords:** high-density lipoprotein, reverse cholesterol transport, HDL therapy, atherosclerosis, CETP

## Abstract

Negative relationship between plasma high-density lipoprotein (HDL) levels and risk of cardiovascular disease (CVD) is a firmly established medical fact, but attempts to reproduce protective properties of HDL by pharmacologically elevating HDL levels were mostly unsuccessful. This conundrum presents a fundamental question: were the approaches used to raise HDL flawed or the protective effects of HDL are an epiphenomenon? Recent attempts to elevate plasma HDL were universally based on reducing HDL catabolism by blocking reverse cholesterol transport (RCT). Here, we argue that this mode of HDL elevation may be mechanistically different to natural mechanisms and thus be counterproductive. We further argue that independently of whether HDL is a driving force or a surrogate measure of the rate of RCT, approaches aimed at increasing HDL supply, rather than reducing its catabolism, would be most beneficial for speeding up RCT and improving protection against CVD.

## A Hero?

Plasma concentration of high-density lipoprotein (HDL) strongly inversely correlates with risk of cardiovascular disease (CVD). This was first convincingly demonstrated in the Framingham Heart Study (1) and confirmed in several large reputable studies (2). Not a single large study presented any evidence contradicting this conclusion or raised doubts in its validity. Inverse relationship between plasma levels of HDL and risk of CVD is as much a medical fact as anything can possibly be.

Considering that the strength and consistency of this relationship is similar to the positive association between low density lipoprotein (LDL) and risk of CVD, and tremendous success of pharmacological treatment of high LDL levels, much effort was applied to repeating the success of statins in a treatment aimed at elevating the HDL. Elevating HDL by lifestyle modifications, such as exercise or dietary intervention, produced positive outcomes, but did not prove the causative role or even a contribution of HDL elevation to their protective effects: the magnitude of changes of HDL level was modest and, more importantly, these interventions affected many other CVD risk factors. Similarly, pharmacological interventions not specifically designed to raise HDL, but associated with higher HDL levels, such as fibrates, were beneficial, but for most of them elevated HDL was only one of the several beneficial effects. A drug specifically designed to elevate HDL level was required to decisively test the hypothesis of benefits of elevating HDL. Several approaches were proposed [for recent reviews see Ref. (3, 4)], and several of them, primarily cholesteryl ester transfer protein (CETP) inhibitors, underwent large-scale phase III clinical trials.

## A Mirage?

The outcome of the trial of first CETP inhibitor, Trocetrapib, was negative: torcetrapib significantly elevated HDL-C levels and reduced LDL-C levels, but total and cardiovascular mortality and morbidity increased (5). The trial of another CETP inhibitor, Dalcetrapib, produced similar outcomes: significant elevation of plasma HDL-C with no effect on cardiovascular mortality and morbidity (6). A genetic study analyzing an association between elevation of HDL-C due to polymorphism in endothelial lipase and risk of myocardial infarction also found no beneficial effect of higher HDL-C levels (7). A trial of niacin, although underpowered to see the effects of actual changes in HDL-C, also suggested a similar conclusion that elevation of HDL-C was not associated with better cardiovascular outcomes (8). What are the possible explanations of an apparent contradiction between clearly cardioprotective effects of naturally occurring high levels of HDL-C, and no such effects of pharmacological elevation of HDL-C?

### Off-target effects

The first explanation brought forward for failure of torcetrapib was its off-target effects. Indeed, patients in the experimental arm of ILLUMINATE study had slightly elevated blood pressure (5). This phenomenon was reproduced in rats, a species that does not express CETP, thus it was clearly an off-target effect (9). However, in addition to a 25% increase in mortality that was observed, this putative off-target effect should also account for lack of 25% decrease in mortality that should have occurred due to lowering of LDL-C level, which did not eventuate, and a 75% decrease in mortality due to elevation of HDL-C, which also did not eventuate. Thus, an off-target effect(s) should have been responsible for about a 125% increase of mortality to offset the expected beneficial effects of treatment if the underlying hypothesis was correct; clearly, a highly unlikely possibility. Finally, another CETP inhibitor, dalcetrapib, did not cause blood pressure elevation, but still did not reduce cardiovascular mortality (6). Overall, given excellent safety record of torcetrapib (10), it is unlikely that failure of CETP inhibitors to improve cardiovascular outcomes was due to off-target effects.

### HDL functionality

Another explanation was that CETP inhibitors elevate plasma level of HDL-C, but impair functionality of HDL. Inhibition of CETP precludes exchange of cholesteryl esters (CE) and triglycerides (TG) between HDL and apoB-containing lipoproteins (mainly Very Low Density Lipoprotein, VLDL) resulting in reduced catabolism of HDL and accumulation of larger TG-rich HDL particles (11). HDL was implicated in many anti-atherogenic activities, and changes in HDL composition or size may affect some aspects of HDL functionality, at least *in vitro*. However, when tested in animals (12) and humans (13–15), this suggestion was not confirmed: HDL functionality, at least toward its two most important functions, reverse cholesterol transport (RCT) and anti-inflammatory properties, was unaffected by inhibition of CETP. Anti-oxidation function of HDL was also unaffected in subjects with CETP deficiency (16). Overall, no evidence was produced to support a hypothesis that failure of CETP inhibitors to improve cardiovascular outcomes was due to impaired HDL functionality.

### Metabolic context

Initial steps of RCT, cholesterol efflux, formation of nascent HDL particles, and early steps of HDL remodeling are very similar in humans and animals. However, late steps of RCT are different: delivery of CE to the liver may occur via direct RCT pathway, selective uptake of CE from HDL by hepatocyte through HDL receptor SR-B1, or indirect RCT pathway, after transfer of CE to VLDL/LDL and uptake of LDL by hepatocyte LDL receptor. The ratio between these two pathways is mainly driven by the activity of CETP and differs between humans and animals.

**Figure 1:**
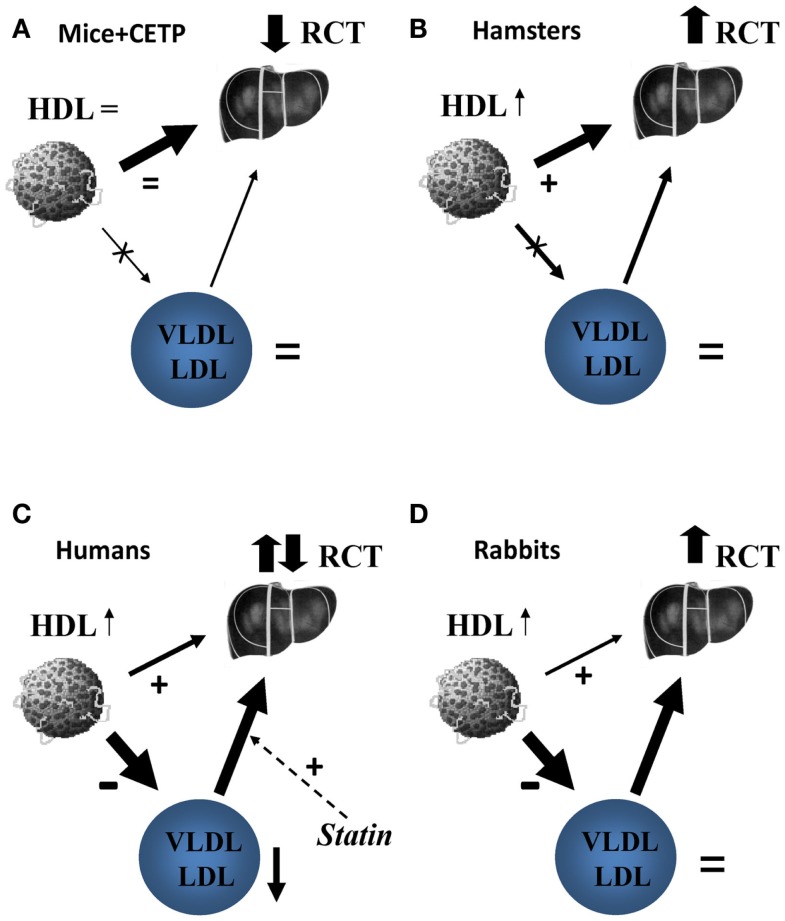
**The proposed effect of CETP inhibition on reverse cholesterol transport in mice (A), hamsters (B), humans (C), and rabbits (D)**. **(A)** In mice, the only pathway of delivery of HDL-C to liver is selective uptake of HDL cholesteryl esters via scavenger receptor type B1 (SR-B1). Introduction of CETP does not affect this pathway, but introduce an additional pathway via apoB-containing lipoproteins and LDL receptors overall increasing RCT. Inhibition of heterologous CETP by Torcetrapib removes this additional pathway and the net effect on RCT is negative. **(B)** In hamsters, the selective uptake of HDL cholesteryl esters by liver is a predominant pathway of reverse cholesterol transport, but the pathway via apoB-containing lipoproteins is contributing. Torcetrapib inhibits the latter leading to increased HDL-C levels that stimulate macrophage cholesterol efflux; the net effect is enhanced reverse cholesterol transport. **(C)** In humans, delivery of HDL cholesterol via CETP and LDL receptors is a major pathway with smaller contribution of direct uptake of HDL cholesteryl esters by liver. Statins stimulate the former pathway by increasing abundance of LDL receptors, torcetrapib inhibits it by blocking CETP. Torcetrapib increases HDL-C, which is associated with an increase in cholesterol efflux and further reduces VLDL/LDL associated with reduced cholesterol efflux. The reverse cholesterol transport under torcetrapib treatment in humans would be a balance between two opposing influences and the net effect is uncertain, likely neutral. **(D)** In rabbits, delivery of HDL cholesterol via CETP and LDL receptors is also a major pathway with minimal contribution of direct uptake of HDL cholesteryl esters by liver. Torcetrapib increases HDL-C, which is associated with an increase in cholesterol efflux, but does not decrease VLDL/LDL levels allowing for transfer of free cholesterol to these apolipoproteins in the course of RCT. The overall balance of reverse cholesterol transport under torcetrapib treatment in rabbits appears to be positive.

Preclinical studies supporting beneficial effects of CETP inhibitors were done in rabbits, a species that, like humans, expresses CETP and is susceptible to diet-induced atherosclerosis (17). HDL metabolism in rabbits, however, is different from that in humans (18). In this regard, it is interesting to compare the effects of CETP deficiency/inhibition in species with different CETP activities (12). Mice do not express CETP, but CETP may be introduced in mice systemically following adenovirus-mediated transfection. Moderate overexpression of CETP in mice did not affect HDL-C or LDL-C levels, but increased the rate of RCT; the effect was blocked by torcetrapib (12). The explanation is likely that supplementation of natural direct pathway with additional indirect pathway resulted in overall higher rates of RCT, and CETP inhibition in this context had an adverse effect on the overall rate of RCT (Figure 1A). Hamsters express CETP, but activity is less than in humans and HDL-C levels are fivefold higher than in humans. Inhibition of CETP in this context resulted in further increase in plasma HDL-C levels and increased the overall rate of RCT (12). The explanation is likely that inhibition of relatively minor indirect pathway and stimulation (through higher HDL-C levels) of the predominant direct pathway resulted in overall faster RCT (Figure 1B). Apparently, in hamsters, liver had sufficient levels of SR-B1 to handle modest amount of additional cholesterol delivered through direct pathway. In humans, CETP activity is high, HDL-C levels are low, and indirect pathway accounts for almost 90% of RCT traffic (19). CETP inhibition would block indirect RCT pathway and increase direct RCT pathway, however, the benefits would depend on a balance between the two and on how much of additional cholesterol flow through direct pathway can be handled by liver (Figure 1C). Does human liver have, or can attain sufficient levels of CLA-1 (human analog of SR-B1) to process sharply increased flow through direct RCT pathway or it gets saturated? Several studies investigated the effect of torcetrapib on fecal cholesterol excretion, the endpoint of RCT, and found no change. In rabbits, CETP activity is triple that of humans and HDL-C level is 15% of that in humans (18). The rate of RCT in rabbits and the effects of CETP inhibitors on this rate were not studied, but the effects on development atherosclerosis were beneficial (17) implying that RCT rate was also elevated (Figure 1D). Interestingly, although torcetrapib caused elevation of HDL-C in rabbits, it was still about half of that in normolipidemic humans and less than a third of what was observed in patients treated with torcetrapib (5), in addition there was no reduction in LDL-C. Perhaps, HDL levels in rabbits, in contrast to humans, are still below saturation of the direct pathway, and rabbits, but not humans, can handle a switch from indirect to direct RCT. One conclusion is, however, clear: the overall effect of CETP inhibition is a balance between inhibition of the indirect pathway and stimulation of the direct pathway of RCT and this balance is greatly influenced by metabolic context.

### Drug interaction

It is important to recognize that in all clinical trials of CETP inhibitors, patients in both arms of the study were receiving statins. Statins inhibit cholesterol biosynthesis in liver causing sharp elevation of the expression of hepatocyte LDL receptors leading to increased catabolic rate of LDL and lowering plasma LDL-C level. Uptake of LDL, however, is also a key step of indirect RCT pathway delivering to liver HDL-derived cholesterol after its transfer to VLDL/LDL. By increasing LDL uptake, statins would stimulate indirect RCT pathway, the very same pathway that is inhibited by CETP inhibitors (Figure 1C). Thus, statins and CETP inhibition have opposing effects on indirect RCT pathway. Furthermore, VLDL/LDL plays an important role in the initial stages of RCT. Most of cellular free cholesterol taken up by HDL ends up in VLDL/LDL prior to esterification, as VLDL/LDL provides a pool with much bigger capacity to hold free cholesterol before relatively slow process of esterification converts cholesterol into esters (20). This finding gave rise to the “shuttle” and “sink” hypothesis (21) also supported by findings that cholesterol efflux to plasma devoid of apoB-containing lipoproteins is much slower and that the availability of apoB-containing lipoproteins may become rate limiting in RCT when HDL-C level are significantly increased (22). Interestingly, humans are the only species where CETP inhibition caused reduction of VLDL/LDL levels (Figure 1), which could also contribute to reductions of overall rate of RCT. In this context, combining CETP inhibitors and statins may have detrimental effect on the actions of both drugs.

## A Witness?

Another possible explanation for the contradiction between inverse relationship between plasma HDL levels and CVD and negative outcomes of the clinical trials aimed at raising HDL is that HDL is not a causative agent at all, but is a biomarker.

Reverse cholesterol transport is essentially a flow taking excessive cholesterol from peripheral cells and tissues to liver for either repackaging or excretion (23). In this context, HDL is an intermediate in this chain of reactions and plasma level of HDL may be a reflection, rather than a driving force of the flow. Static level of an intermediate, however, is a good marker of the flow rate if the rate-limiting step is located before a particular intermediate, e.g., generation of HDL through cholesterol efflux. If the rate-limiting step is located after this step, e.g., catabolism or remodeling of HDL, then high level of the intermediate may be caused by its inability to proceed along the pathway and indicates a retarded rather than accelerated flow. In this context, attempts to elevate HDL-C levels by inhibiting its remodeling, as it happened with CETP inhibitors, niacin and polymorphism in endothelial lipase, may reduce the flow through RCT and are counterproductive. Approaches aimed at increasing supply of HDL, such as infusion of rHDL, may therefore be more productive in increasing RCT and higher levels of HDL achieved in this manner may be more reflective of RCT flow rate.

Furthermore, in the context of “flow” concept, static plasma HDL level may also reflect the rate of HDL formation, and therefore, the activity of the main cholesterol transporter responsible for the HDL formation, ABCA1 (24). ABCA1, however, is not only a key molecule in HDL formation, but also a key regulator of cholesterol efflux, a process removing excessive cholesterol from cells, including vascular cells. Consequently, HDL-C may be a true biomarker of ABCA1 activity, and it is ABCA1 activity that protects against cholesterol accumulation and risk of CVD, and ABCA1 should be a target for treatment, rather than HDL level.

Admittedly, the role of HDL in RCT is not the only atheroprotective function of HDL. HDL also displays anti-inflammatory, anti-oxidant, anti-platelet, anti-apoptotic, and other properties that should be beneficial for reducing cardiovascular risk [for review, see Ref. (25)]. The dependence of these properties on the role of HDL in cellular cholesterol metabolism and systemic RCT is unclear, as is the contribution of each of these properties to overall cardiovascular protection associated with higher HDL levels. However, assuming that these properties contribute to overall atheroprotection and are independent of cholesterol metabolism, it is possible that raising HDL by any means would be beneficial, but so far there is little evidence that this is the case.

## The Light at the End of the Tunnel or the Headlamp of an Oncoming Train?

Negative outcomes of large clinical trials of HDL-targeted therapy prompted several large pharmaceutical companies to abandon further attempts to develop approaches to elevate plasma HDL and declare the death of HDL Therapy. However, the negative relationship between naturally occurring plasma HDL-C levels and risk of CVD has never been challenged and the issue is whether pharmacological elevation of plasma HDL would have the same protective effect. The obvious question is then whether the mechanisms responsible for naturally high HDL levels were the same mechanisms targeted in attempts to raise HDL pharmacologically. Both mechanisms are grossly under investigated, but within this limitation the answer is probably not. Targeting HDL catabolism increases HDL-C levels, but at the same time retards RCT and may have the outcome opposite to what was intended. Alternative approaches, such as increased supply of HDL, may be more appropriate. Thus, the outcomes of several human trials of rHDL infusion were encouraging (26, 27). RVX-208, an epigenetic inducer of biosynthesis of apolipoprotein A-I, produced positive outcomes in preclinical studies (28), but reportedly was toxic in humans. Negative outcomes of clinical trials created understandable skepticism, but there are too many questions about validity of the approaches tested in these trials to provide a credible challenge to the idea of HDL-targeted therapy. With better understanding of how HDL and RCT protect against CVD, the last word in HDL story is yet to be written.

## Conflict of Interest Statement

The author declares that the research was conducted in the absence of any commercial or financial relationships that could be construed as a potential conflict of interest.
